# SE-COTR: A Novel Fruit Segmentation Model for Green Apples Application in Complex Orchard

**DOI:** 10.34133/plantphenomics.0005

**Published:** 2022-12-15

**Authors:** Zhifen Wang, Zhonghua Zhang, Yuqi Lu, Rong Luo, Yi Niu, Xinbo Yang, Shaoxue Jing, Chengzhi Ruan, Yuanjie Zheng, Weikuan Jia

**Affiliations:** ^1^ School of Information Science and Engineering, Shandong Normal University, Jinan 250358, China.; ^2^ State Key Laboratory of Biobased Materials and Green Papermaking, Qilu University of Technology (Shandong Academy of Science), Jinan 25035, China.; ^3^ School of Informatics, University of Leicester, Leicester LE1 7RH, UK.; ^4^ Department of Engineering Design and Mathematics, University of the West of England, Bristol BS16 1QY, UK.; ^5^ Fujian Key Laboratory of Intelligent Control and Manufacturing of Agricultural Machinery, Wuyishan 354300, China.; ^6^ Key Laboratory of Facility Agriculture Measurement and Control Technology and Equipment of Machinery Industry, Jiangsu University, Zhenjiang 212013, China.

## Abstract

Because of the unstructured characteristics of natural orchards, the efficient detection and segmentation applications of green fruits remain an essential challenge for intelligent agriculture. Therefore, an innovative fruit segmentation method based on deep learning, termed SE-COTR (segmentation based on coordinate transformer), is proposed to achieve accurate and real-time segmentation of green apples. The lightweight network MobileNetV2 is used as the backbone, combined with the constructed coordinate attention-based coordinate transformer module to enhance the focus on effective features. In addition, joint pyramid upsampling module is optimized for integrating multiscale features, making the model suitable for the detection and segmentation of target fruits with different sizes. Finally, in combination with the outputs of the function heads, the dynamic convolution operation is applied to predict the instance mask. In complex orchard environment with variable conditions, SE-COTR achieves a mean average precision of 61.6% with low complexity for green apple fruit segmentation at severe occlusion and different fruit scales. Especially, the segmentation accuracy for small target fruits reaches 43.3%, which is obviously better than other advanced segmentation models and realizes good recognition results. The proposed method effectively solves the problem of low accuracy and overly complex fruit segmentation models with the same color as the background and can be built in portable mobile devices to undertake accurate and efficient agricultural works in complex orchard.

## Introduction

Fruit harvesting is a time-consuming yet far-reaching task. As the demographic aging is becoming more severe, the increasing cost of labor is seriously limiting the development of agricultural products on a larger scale. Therefore, automated harvesting robots that can perform automatic operations in the field are emerging as a promising technology for future agricultural development. Unlike conventional automated harvesting of crops, automated harvests of fruits such as green apples are at a further complex situation [1]. Vision systems are required for fruit detection and localization for robotic fruit harvesting. Machine vision technology was first successfully applied in remote sensing and biomedical image processing and progressively extended to many fields such as aerospace, industrial inspection, and intelligent agriculture. In the production management of fruit and vegetable industry, machine vision has the advantages of efficient and accurate fruit detection and segmentation and has gained extensive research and attention from scholars in recent years [2].

Designing vision systems aiming at fast recognition and accurate segmentation is a demanding task. Accurate segmentation of green fruit is the core of machine vision, which has great application value and practical importance for realizing automated applications such as machine picking, pest and disease early warning, field fertilization, and yield prediction. Fast and accurate segmentation application of green target fruits in the complex orchard environment is a very challenging problem for developing harvesting robots and faces many difficulties. In terms of segmentation performance, there are many disturbing factors in the intelligent operation that will affect the accuracy and efficiency of the model under natural orchard conditions, such as overlapping fruit shadows, multiple lighting, and multiple angles. Especially, the color features of green apples are similar to the background color of branches and leaves, which are more difficult to be distinguished and recognized.

In the early intelligent agriculture, traditional machine learning methods are first used to recognize and locate target fruits by extracting feature information like chromatic, morphological, or textural information from images [3]. By combination of partial image characteristics and chromatic information, Fan et al. [4] presented a pixel patch segmentation approach on the basis of gray-centered RGB color space to quickly and accurately recognize apple targets in unstructured natural orchards under illumination conditions. Wang et al. [5] proposed a new unsupervised density peaking clustering algorithm for green apple segmentation, which improves the segmentation accuracy and the fitting effect of fruit boundaries compared with other clustering algorithms. Liu et al. [6] designed an approach by block categorization to recognize and segment apples in plastic bags, and the watershed algorithm was utilized to divide images into blocks. The model can effectively suppress the effect of light. Kang and Chen [7] explored a network for real-time detection and segmentation of fruits in an orchard environment. The feature extraction of the model is achieved by an atrous spatial pyramid pooling and a gated feature pyramid network (FPN). Traditional vision methods depend on hand-crafted characteristics to extract distinguishing information, which has shortcomings in terms of accuracy, robustness, and efficiency in real-world work environments. Because of overlapping occlusions, lighting changes, and complex backgrounds, traditional target recognition algorithms have difficulty detecting apples in natural environments. With high complexity and low robustness, they are difficult to adapt to the complex and unstructured orchard environment.

In contrast, deep learning is one of the latest modern techniques for image processing and data analysis with good results and great potential. Nowadays, deep learning has been adopted to automatically extract features such as pixels, position, and scale of apples from images, bringing a new perspective to object fruit detection and segmentation [8,9]. Object detection and segmentation techniques based on deep learning have been shown to achieve higher robustness and accuracy in recognizing objects in natural environments [10,11]. Bargoti and Underwood [12] first used multiscale multilayer perceptron and convolutional neural network (CNN) for feature learning, followed by fusion of watershed segmentation and circular hough transform algorithms to achieve pixel-level segmentation output of target fruits. Kim et al. [13] developed a deep learning-based fruit tree segmentation model and generated an intelligent spraying system for fruit trees, which obtained satisfactory performance. Jia et al. [14] refined mask region CNN (Mask R-CNN), which is more applicable to the localization and recognition of overlapping apples. Liu et al. [15] built a two-layer segmentation network to handle shading and shaded target fruits separately in the natural orchard environment. Jia et al. [16] proposed a robust segmentation network (RS-Net) by extending Mask R-CNN for fruit production. RS-Net outperformed other advanced models for segmentation of overlapping apples. Li et al. [17] constructed a new ensemble U-Net green apple segmentation algorithm utilizing atrous spatial pyramid pooling method under complex orchard environments, which markedly contributed to accuracy and adaptability of the model for segmenting green fruits. In addition, segmentation methods by locations [18,19] directly segment instances by classifying each grid and performing end-to-end mask prediction. However, when the fruit background is overly complex and the targets are overly dense, it is inevitable to waste a lot of computational effort and time on the background pixels. The above deep learning algorithm-based detection and segmentation models have greatly improved in accuracy and generalization ability, outperforming traditional machine learning methods and effectively reducing computational complexity.

As deep learning continues to evolve, the attention mechanism [20] has become the focus of research in many computer vision applications, such as image classification [21] and image segmentation [22], and often embedded in the backbone network of the model to assist in more efficient feature extraction tasks. Because of the ability to construct a spatial or channel attention, the self-attention mechanism was initially used in transformer for natural language processing and has become very popular in recent years. However, the self-attention module is often used in large models [23,24] and is not applicable to mobile networks owing to its high computational effort in time and memory and its impact on the adaptability of the model in different environments.

With the above advances, simple yet efficient fruit harvesting in real orchard environments is still a huge challenge. Segmenting objects by transformers [25] is a hybrid instance segmentation model based on twin transformers, first dividing the input feature map into small patches and then predicting the class of each patch while dynamically segmenting each instance, which combines CNNs and transformers to extract features. Although segmentation mode is relatively simple, the segmentation effect for green fruits needs to be improved because of the high complexity and variability of the environment in which the green apples are located.

Some state-of-the-art segmentation models like Mask R-CNN are two-stage models constructed by directly adding mask branches behind the object detectors like Faster R-CNN, which are computationally intensive and complex. These methods focus on performance over speed, which cannot meet the requirements of real complex orchard environments for robots to work efficiently in real time. The aim of this study is to develop a novel green fruit segmentation model to get better segmentation effect and support the real-time operation of the harvesting robot in the natural orchard. Inspired by segmenting objects by transformers, we rethought fruit segmentation steps and innovatively built a simple and efficient segmentation model targeting green fruits, termed SE-COTR (segmentation based on coordinate transformer), which tackles the problem of accurate and real-time segmentation of green apples by harvesting robots in complex orchard environments. Our main idea is to perform accurate and efficient fruit segmentation by maintaining the power of the current two-stage approach in predicting instances and avoiding causing higher computational complexity in the segmentation process.

Specifically, the whole network framework includes four parts. First, a lightweight backbone network MobileNetV2 [26] is used to connect the FPN [27] together for extracting and fusing image features to generate multiscale feature maps. Second, COTR module with the coordinate attention mechanism [28] is constructed for modeling global and semantic dependencies and outputting multidimension sequence features for subsequent prediction, enhancing the focus on valid features. Next, inspired by FastFCN [29], joint pyramid upsampling (JPU) is used to merge the features of the FPN layer with those of COTR into a unified mask feature. Finally, on the basis of the outputs of the function heads, the dynamic convolution operation is used to generate the instance mask. The model is developed on the basis of the constructed green apple dataset to obtain 61.6% segmentation accuracy with less floating point of operations (FLOPs), which has surpassed the current popular segmentation models regarding real-time performance. It can adapt to the unstructured natural orchard environment with good robustness.

Overall, this study includes the following main contributions:

1. A novel and efficient green fruit segmentation model SE-COTR is proposed, using a lightweight network MobileNetV2 for feature extracting, which is flexible and efficient to be easily inserted into the mobile networks.
2. COTR module is constructed for enhancing attention to valid features and modeling global and semantic dependencies, with obvious computational and memory savings.
3. JPU method is employed to fuse the features of each FPN layer and COTR into a uniform mask feature for subsequent mask generation.
4. The proposed SE-COTR outperforms the advanced models with regard to complexity and accuracy, which is more targeted and effective in detecting and segmenting green apples in complex orchard environments.

The other sections in this work are arranged in the following. Section 2 describes the data acquisition and the relevant dataset processing. Section 3 designs a novel green fruit segmentation model with effective modules. Section 4 shows the related experiments and makes a comparison of the segmentation effect with popular models. At last, section 5 discusses the presented method and stands for further research directions for green fruit detection and segmentation.

## Materials

### Data acquisition

For this study, we labeled and generated a valid green apple dataset by collecting rich image data in real orchards to facilitate the construction of the model.

Taking immature Gala apples as the object, we conducted fruit segmentation study for green apples. Since there were few publicly available green fruit datasets and the image states were not eligible, we collected images of fruits in natural orchards in different states to produce a dataset in a certain format.

The collection sites are in the South Mountain District, Jinan, and Longwang Mountain Apple Production Base in Fushan District, Yantai City, Shandong Province.

**Fig. 1.**
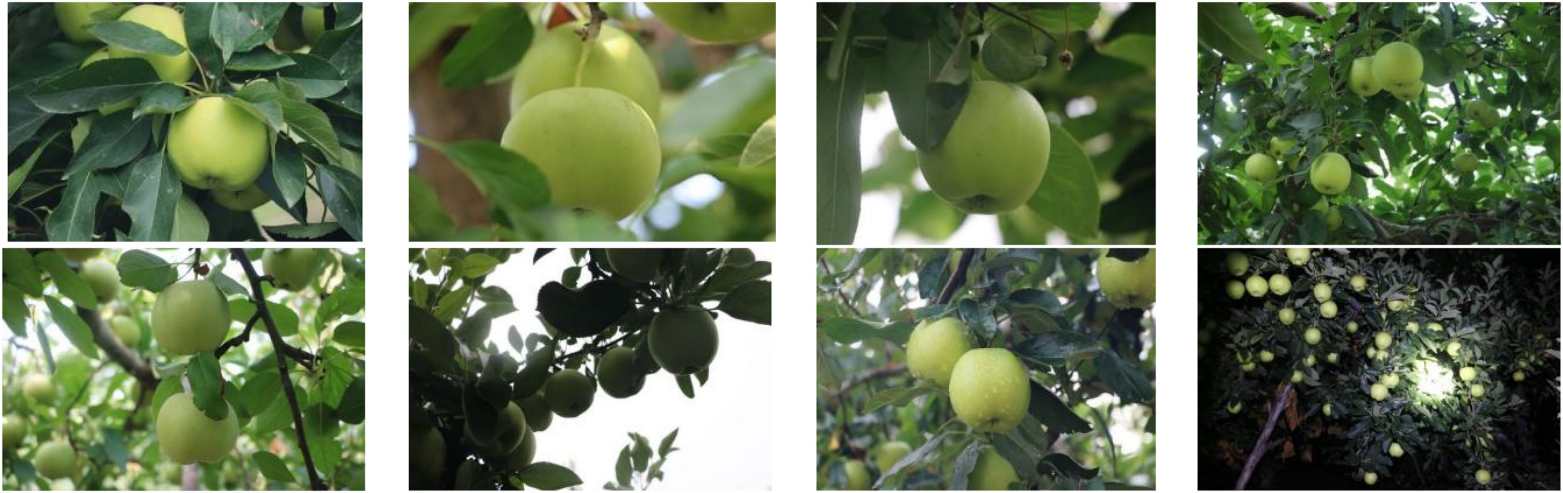
Apple images from the complex orchard.

In the unstructured orchard, there are various irresistible natural factors such as overlapping, obscuring, droplets of water, and varied lights at different angles. Considering the operational scenarios of orchard yield measurement and automatic machine picking, the image acquisition was as comprehensive as possible. Different camera positions were set to capture multiple angles around the fruit trees to acquire images of the target fruit in different poses. The illumination included natural light during the daytime and light-emitting diode-assisted light sources in the evening. Figure 1 shows some examples of apple images collected in different scenarios.

We capture 468 apple images with the resolution of 6,000 × 4,000 using Sony Alpha 7 II camera. To facilitate the computation of deep learning networks on this dataset and meet the fast and effective segmentation requirements of the intelligent robots in the orchard, we harmoniously resize the images to 600 × 400.

During the target training process, overfitting of deep learning networks occurs when the training dataset is too small, which reduces the generalization of the network. To overcome this problem, we perform data augmentation on the collected images, which can also minimize the unbalanced distribution of training samples. The number of images in the dataset before and after data augmentation is 468 and 1,386, respectively. Methods of data enhancement consist of geometric and photometric changes. For geometric distortion, we perform random shifting, flipping, and rotation. For photometric distortion, we adjust the brightness, contrast, and saturation of the image. The images are randomly augmented and enhanced according to a certain probability.

### Dataset production

**Fig. 2.**
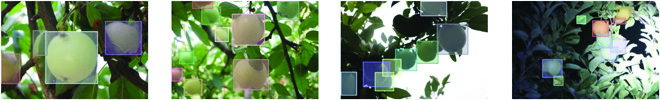
The result of fruit visualization with annotation information.

Low-quality images with unclear features are removed, and the remaining 1,361 apple images were used to label and make the dataset. Ground truth labeled is manually generated for each image, containing the individual segmentation of all the apples shown in the image. LabelMe software can precisely outline each visible fruit in the image and produce the mask. The rectangular area is the range of the target fruit area. The bounding box is then automatically calculated on the basis of the irregular contours of the fruit, saving processing time. Each JSON file is a one-to-one correspondence with the original apple image, containing the target annotation information of the original image, and is finally converted into a COCO format dataset. The result of fruit visualization with annotation information is shown in the Fig. 2.

## Methods

A novel fruit segmentation model SE-COTR is built to achieve accurate and fast green fruit segmentation, without first detecting but directly reaping the segmentation mask of the object. Unlike typical segmentation models, the proposed SE-COTR focuses on segmenting target instances directly in the absence of box detection, greatly simplifying the whole process, while ensuring an optimal balance between speed and accuracy of the fruit segmentation model leveraging the coordination between multimodules.

**Fig. 3.**
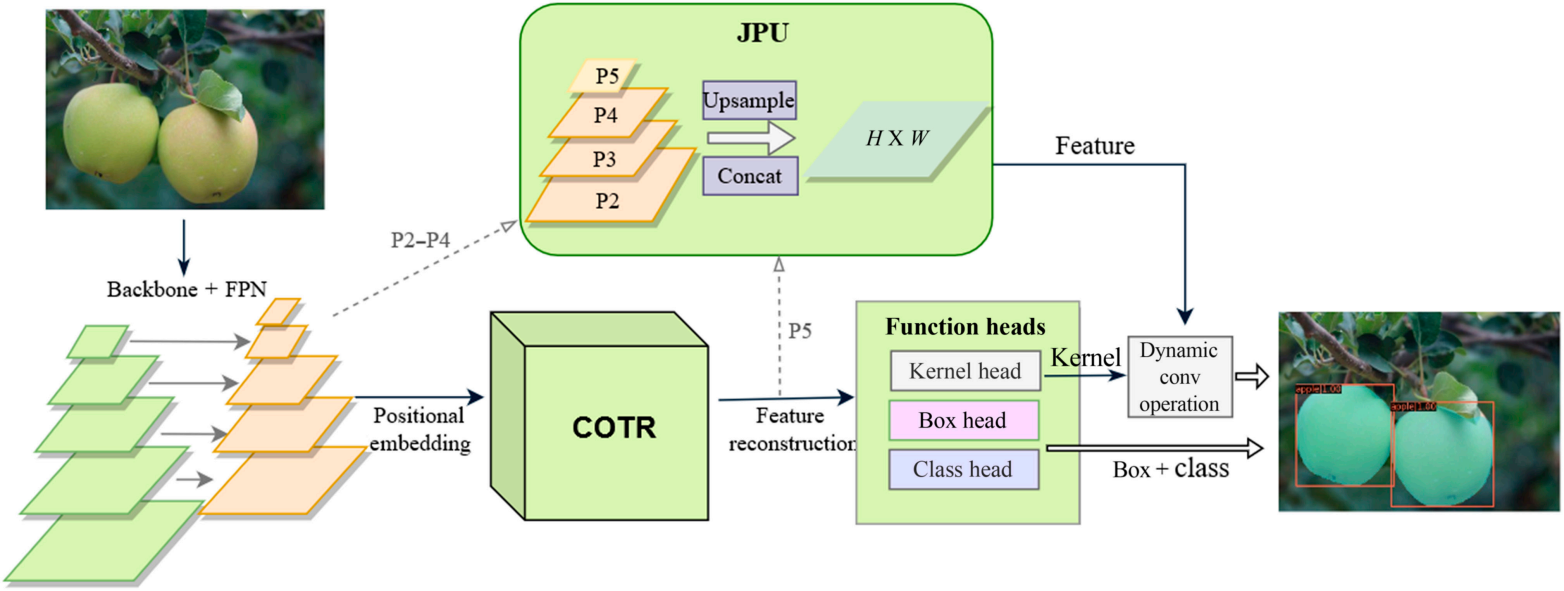
SE-COTR network architecture.

The whole structure of SE-COTR is depicted in Fig. 3, and innovations are highlighted in green. Specifically, the lightweight backbone network MobileNetV2 is connected with the FPN for sufficiently extracting image features and generating multiscale feature maps without too much computational workload. In this way, the features of the input image, especially the low-level and local features, are fully extracted. The whole extracted features are fed to the COTR for modeling global and semantic dependencies and output multidimensional sequence features for subsequent prediction in different functional heads. The P2 to P4 features in FPN and P5 from COTR are input to the JPU, which fuses the multiscale features into a unified scale feature through the upsampling and cascading operations. Finally, the generated feature mappings and the corresponding convolution kernel are utilized to produce target masks through the dynamic convolution operation to achieve accurate green fruit segmentation.

### Coordinate transformer

Fruit overlapping, branches and leaves shading, and variable lighting lead to difficulties in detecting and segmenting green apples. The attention mechanism enables the model to focus on and enhance the effective feature information while ignoring some useless information, thus improving the robustness and stability of the model to environmental changes in unstructured orchards. In addition, considering that positional information is crucial for producing spatially selective attention graphs in mobile networks, we employ the coordinate attention mechanism that can effectively capture location information and channel relationships.

First, coordinate attention catches the relationship between channels and captures orientation-aware and position-sensitive information. This can strengthen the feature representation of the mobile network and help the model to more accurately locate and recognize objects of interest. Second, the coordinate attention is flexible and lightweight and easy to be embedded in the mobile network building blocks, so we apply it to build the COTR module of the proposed model. Compared with the original transformer, the proposed COTR has marked computational and memory savings, especially for dense prediction cases such as instance segmentation. The details are shown below.

#### Coordinate attention module

The coordinated attention module can be considered as a computational unit whose purpose is to improve feature representation in mobile networks and is a lightweight attention suitable for mobile networks. Coordinated attention effectively encodes channel relations and long-range dependencies through two processes. Sequentially, coordinate information embedding and coordinate attention generation are performed.

To motivate attention blocks to exploit accurate location information to spatially catch remote interactions, the two-dimensional global pooling is decomposed into two one-dimensional feature encoding operations. Specifically, for an input X, each channel is first encoded along the horizontal and vertical coordinates using a pooling kernel of size (*H*,1) or (1,*W*), respectively. Thus, the output of the *c*th channel with height *h* and width *w* are respectively represented as:

$$z_{c}(h)=\frac{1}{W}\sum_{0\leq i\leq W}x_{c}(h,i)$$ (1)

$$z_{c}(w)=\frac{1}{H}\sum_{0\leq j\leq H}x_{c}(j,w)$$ (2)

where *h* is the length of the feature map, *w* is the width of the feature map, *c* is the number of channels in the feature map. *z_c_*(*h*)channel for feature encoding along the height *h* coordinate, and *z_c_*(*w*) is the output along the weight *w* coordinate. *x_c_*(*h*,* i*) and *x_c_*(*j*,* w*) are the input features positioned at certain height *h* coordinate and weight coordinate *w* in the *c*th channel.

With the above two transformations, coordinate attention decomposes channel attention into two one-dimensional feature encoding processes that assemble features at two different spatial axes. In this way, remote dependencies are captured along one spatial direction, while accurate location information is reaped and maintained along the other direction, allowing the network to locate targets more accurately.

Given that the feature map of each layer of FPN is *H* × *W* × *C*, the feature map is first divided into *N* × *N* patches *P* ∈ *R*^*N*×*N*×*C*^, which are stacked as fixed blocks in horizontal and vertical directions. The location embedding is inserted into the block to maintain the location information. The resulted feature maps are then encoded to generate a pair of orientation-aware and location-sensitive attention maps that are used to strengthen the focus of objects.

According to above transformations, the global receptive field can be well reaped, and the precise position information can be encoded. To utilize the generated representations, which provided the integrated feature maps generated by the information embedding transformations of Eqs. 1 and 2, they are first concatenated and then transformed using the shared 1 × 1 convolutional transform *F*_1_ function:

$$f=\delta (F_{1}([z^{h},z^{w}]))$$ (3)

Among them, *f* represents the intermediate feature map, which is responsible for encoding the information in both spatial directions. [∙,∙] indicates the cascaded operations in spatial dimensions, δ represents a nonlinear activation function, and *f* is decoupled into two independent tensors *f^  h^* and *f^ w^* based on the spatial dimension.

Following that, two other 1*1 convolutional transform functions *F_h_* and *F_w_* are utilized to convert *f^  h^* and *f^ w^* into output tensors *g^ h^* and *g^ w^* with the equivalent number of channels, respectively, i.e.

$$g^{h}=\sigma \left(F_{h}\left(f^{h}\right)\right)$$ (4)

$$g^{w}=\sigma \left(F_{w}\left(f^{w}\right)\right)$$ (5)

where *σ* is a Sigmoid function. We take the extended outputs *g^ h^* and *g^ w^* to be attention weights, respectively.

As a final step, coordinate attention block is generated according to the following equation:

$$y_{c}(i,j)=x_{c}(i,j)\times g_{c}^{h}(i)\times g_{c}^{w}(i)$$ (6)

where *y_c_* is the output of channel *c* of the coordinate attention block for exact position.

Coordinate attention focuses both horizontally and vertically for the input tensor, with each element reflecting the presence or absence of an object in both columns and rows. By encoding the information, coordinate attention can more precisely pinpoint the specific location of targets, which enhances the convolutional feature representation of the mobile network and makes the whole model work better.

#### COTR layer

**Fig. 4.**
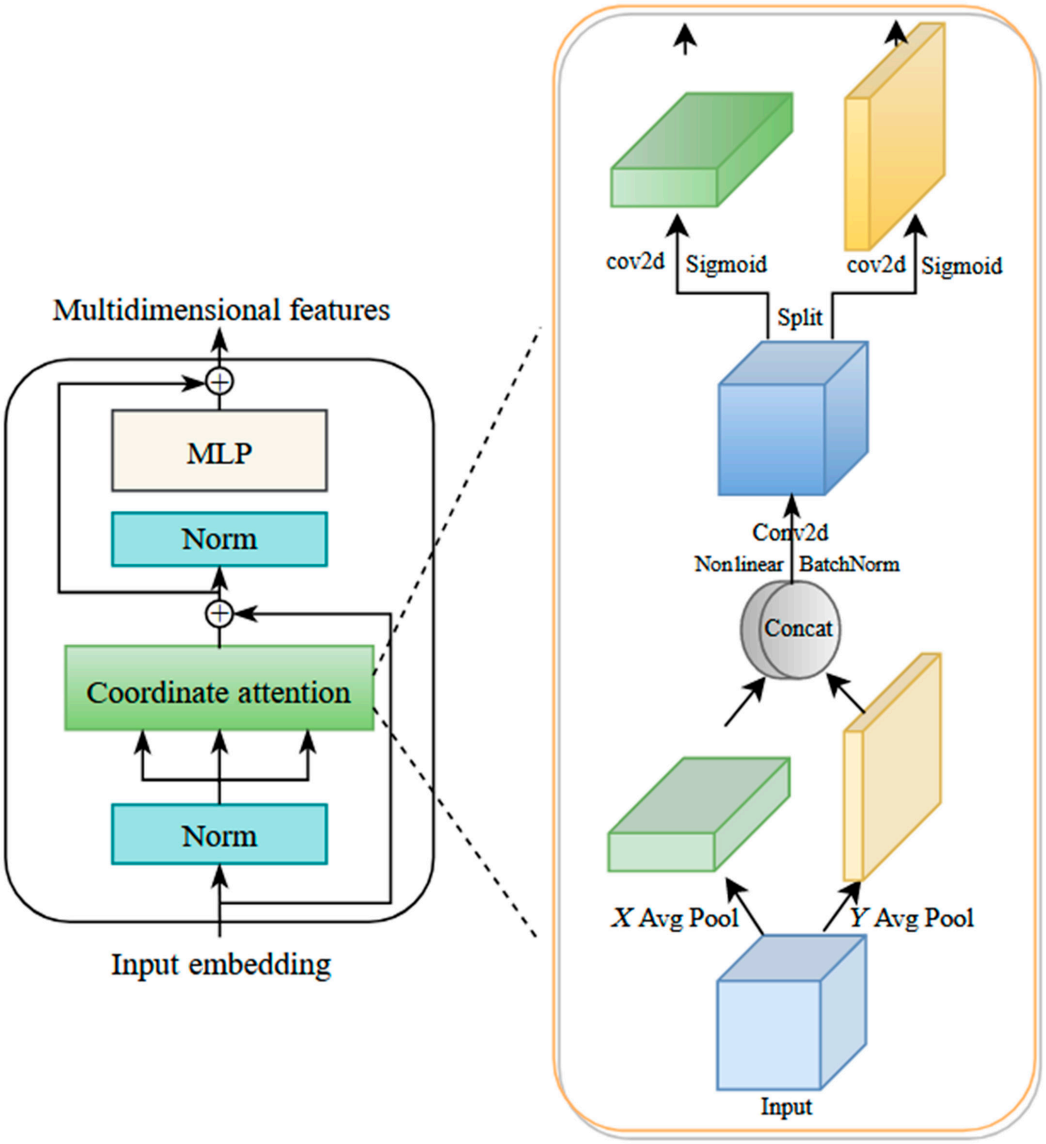
Flowchart of COTR layer.

The original transformer layer is similar to the encoder used in natural language processing and consists of two parts, a normalized self-attention mechanism and a multilayer perceptron after layer normalization, and these two parts are connected with residuals [30]. Finally, the multidimensional sequence features are reaped as the output connections of the *k* transformer layers for successive prediction in multifunction heads. As a way to strike a better balance between computational complexity and feature extraction effectiveness, we applied the original transformer layer design and constructed the COTR layer by only replacing the attention with coordinate attention, as shown in Fig. 4.

### Joint pyramid upsampling

To reap the characteristic representation of mask for location-sensitive and instance-level segmentation, an easy way is to predict the feature map for each different scale, but it increases time and resources. With JPU, multiscale context information can be exploited across multiple layers of feature maps, which will result in improved performance. For the objective of reducing complexity, we treat the process of reaping high-resolution feature maps as a joint upsampling issue. Hence, JPU module is used to combine the features of each FPN layer and COTR into a unified mask feature.

**Fig. 5.**
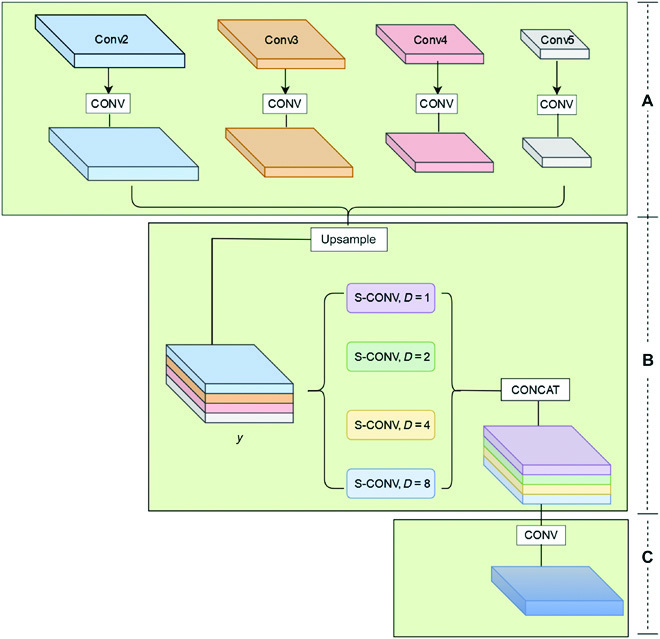
JPU module.

The module takes the four feature maps (Conv2 to Conv5) as input. Specifically, each input feature map is first mapped into the same space with regular convolution blocks (Fig. 5A), leading to better fusion and less computational complexity. After that, the resulting feature maps are upsampled and concatenated to produce *y* (Fig. 5B). Different expansion rates (1, 2, 4, and 8) are assigned to four separable convolutions to simultaneously extract features from *y*. Eventually, the features are transformed into a final prediction by an additional convolutional block (Fig. 5C). JPU module is able to jointly solve two closely related JPU issues, upsampling Conv3 based on Conv2, upsampling Conv4 based on Conv3, and upsampling Conv5 under the guidance of an amplified Conv4, respectively. Therefore, JPU is capable of extracting contextual information at different scales from multilevel feature maps, which results in better performance.

The P2 to P4 features from the FPN are fused with P5 of location information from the COTR, and the input features at each scale are processed. Different multiples of bilinear upsampling are performed for P3 to P5, in order to achieve a resolution of $\left(\frac{H}{4},\frac{W}{4}\right)$. After processing, P2 to P5 are then cascaded together after JPU to generate the final unified *H* × *W* feature map.

### Function heads for Mask prediction

The features of the COTR module are fed to the function heads to perform subsequent predictions. Function heads include three parallel head functions, the kernel head branch, the box head branch, and the class head branch.

**Fig. 6.**
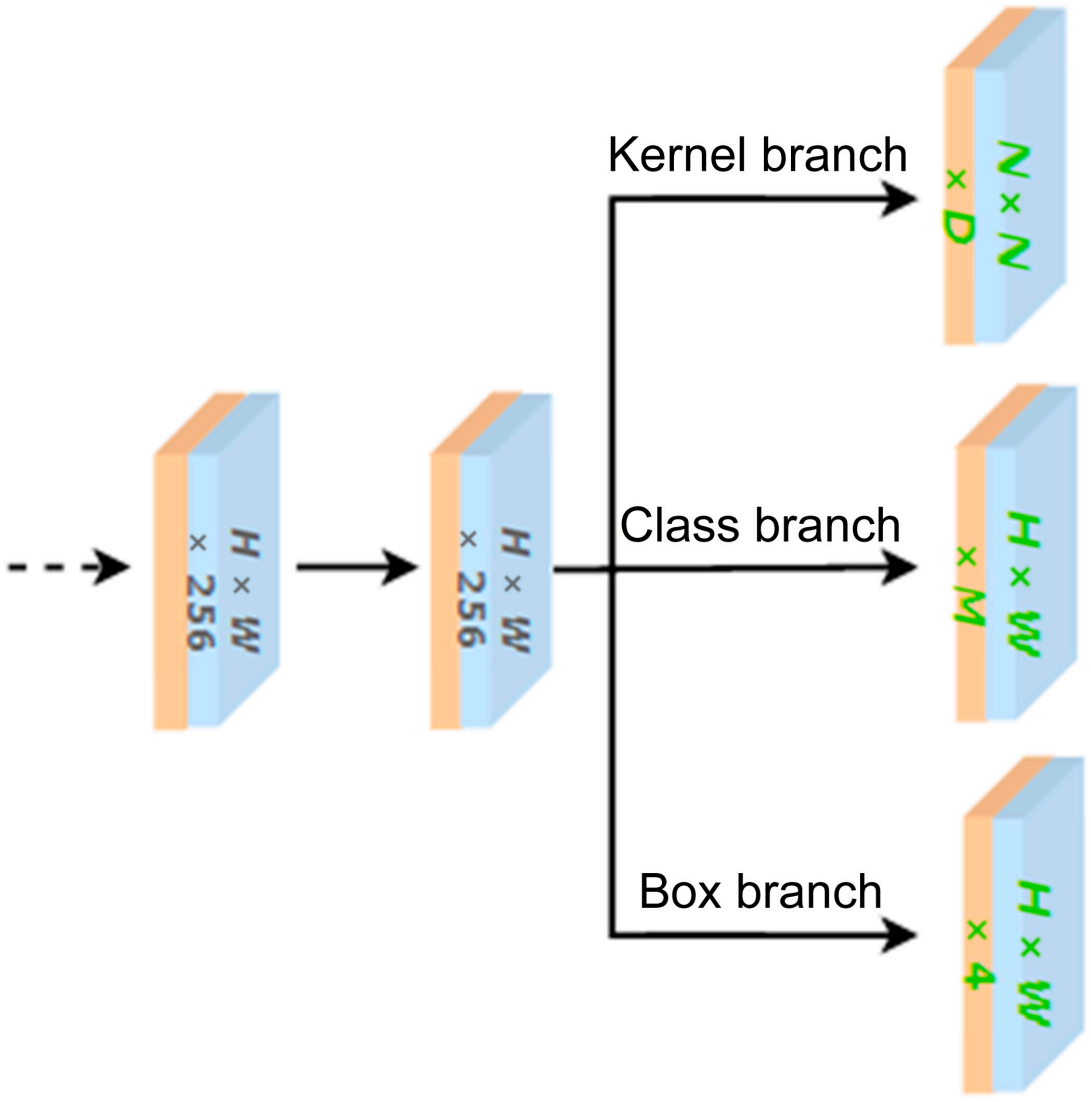
Three parallel head branches in function heads.

The convolutional kernel head consists of a linear layer that outputs a *N* × *N* × *D* tensor in parallel with the class head, in which the tensor represents *N* × *N* convolutional kernels with *D* parameters. The class head also contains a linear layer for outputting an *N* × *N* × *M* classification consequence, where *M* is the amount of classes. As a single class is assigned to each patch for a single target whose center falls inside, such as YOLO (you only look once) [31], multilayer prediction is employed to share the heads at different feature layers, further improving the efficiency and performance of the model for multiscale target fruits. In the box prediction branch, the downsampling operation is used to map the positive sample coordinates onto the input image, and then the normalized offset between the projected coordinates and the four edges of the ground-truth box G is computed to get the regularized target. For the sake of clarity and simplicity, the three parallel head branches are integrated together, as shown in the Fig. 6.

During training, focal loss is used for computing the classification loss, Smooth L1 loss function is employed to train the bounding box loss, and all oversight of those convolution kernels is derived from the final mask loss.

For an instance mask prediction, the proposed model SE-COTR performs a dynamic convolution operation on a uniform feature map to generate a mask for each patch. Given the predicted convolutional kernels *K* ∈ *R*^*N*×*N*×*D*^ from the kernel head, each kernel is in charge of generating masks for the instances in the corresponding patch. The specific operation can be expressed as follows.

$$Z^{H\times W\times N^{2}}=F^{H\times W\times C}*K^{N\times N\times D}$$ (7)

where * represents the convolution operation. *Z* is the final generated mask in one dimension of*H* × *W* × *N*^2^. The value of *D* depends on the shape of the convolution kernel, i.e., *D* = *γ*^2^*C*, where *γ*is the size of kernel. The eventual segmentation masks are produced by Matrix nonmaximum suppression, and each of them is separately supervised by Dice Loss [32].

## Results

For the green apple dataset, abundant experiments were conducted on ablation and comparisons in order to ensure the training accuracy and efficiency of the model, and the results were assessed in terms of standard metrics such as mean average precision (mAP) and parameters.

### Experiment setting

The experiments were performed on Ubuntu 18.04 operating system, with 32-GB graphic processing unit (GPU) Tesla V100 and v10.1 CUDA environment. All procedures were written in Python 3.7 on the PyTorch framework. In the experimental setup, the constructed model is to be pretrained on the publicly available COCO dataset [33] first to expedite the whole training process and reduce the model computation. The COCO dataset is a widely used dataset for computer vision released by Microsoft. The dataset includes 200,000 labeled images with a total of 80 classes for initial validation of model performance. Then, the model is trained on the created green apple dataset in the complex environment, and the parameters are fine-tuned to attain the optimal network.

The apple dataset is split into two subsets for training and validation at a ratio of 7:3, and the training and validation subsets have no intersection. The green apple dataset is split into two main parts: datasets_training and datasets_validation. Training is performed on datasets_training, and testing and evaluation are performed on datasets_validation. The training set consists of 953 images and the validation set consists of 408 images. The details of images and instances on the divided apple dataset are shown in Table 1.

**Table 1.** The numbers of images and instances on the divided apple dataset.

**Dataset**	Num_images	Num_instances
Training set	953	4,943
Validation set	408	2,194
Total	1,361	7,137

In the training dataset, to ensure the validity of the comparison test, we used a stochastic gradient descent to train the model. Synchronous stochastic gradient descent was used on 2 GPUs, with each GPU processing 8 images. In addition, the model is trained for 24 epochs at an initial learning rate of 0.0025. The weights are decayed to 0.0001 and the momentum is 0.9. Next, the top 100 score predictions for each image are selected by applying a nonmaximum suppression with a threshold of 0.5. No other tricks are utilized to maintain simplicity.

### Evaluation metrics

Four typical metrics, precision (*P*), recall (*R*), *F*_1_ score (*F*_1_), and mAP, were used to evaluate the performance of the model in segmenting target apples. *F*_1_ score is a balanced *F* score defined as the summed average of precision and recall. *F*_1_ score combines the results of precision and recall outputs. The values from 0 to 1 correspond to the worst output to the best output of the model. Some metrics are defined below.

$$P=\frac{\mathrm{TP}}{\mathrm{TP}+\mathrm{FP}}$$ (8)

$$R=\frac{\mathrm{TP}}{\mathrm{TP}+\mathrm{FN}}$$ (9)

In the equations, *TP* indicates the true positives, *FP* represents false positives, and *FN* stands for false negatives. The synthesis metric *F*_1_ is shown in the Eq. 10.

$$F_{1}=\frac{2\mathrm{PR}}{P+R}$$ (10)

**Fig. 7.**
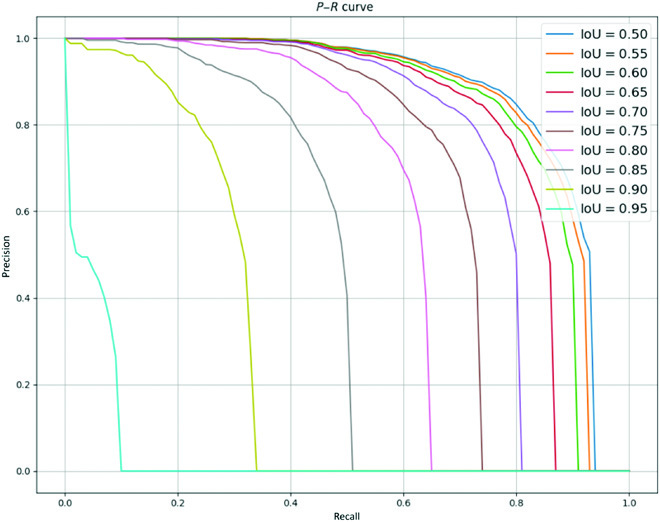
*P*–*R* curves under 10 different IoU thresholds.

To better assess the accuracy of the overall model, the average precision (AP) of the model at each pixel-wise Intersection over Union (IoU) value in [0.5, 0.55, 0.6, …, 0.95] is calculated and averaged to obtain the comprehensive indicator mAP, which combines the accuracy and recall of the model, as follows.

$${\mathrm{AP}}_{\mathrm{IoU}=i}=1/101\sum_{r\in R}p\left(r\right)$$ (11)

$$m\mathrm{AP}=1/10\sum_{i\in I}{\mathrm{AP}}_{\mathrm{IoU}=i}$$ (12)

where *i* is the IoU threshold in [0.5, 0.55, 0.6, …, 0.95]. *r* means recall. *R* is [0, 0.01, 0.02, ..., 1.0] with 101 values and an interval of 0.01, which can be estimated as the area of the precision–recall (*P*–*R*) curve under the specified IoU threshold. For 10 different IoU thresholds, the precision–recall (*P*–*R*) curve is shown in Fig. 7.

Furthermore, the following indicators are also utilized:

• *AP*_50_ and *AP*_75_: The *AP* value as IoU is set to 0.5 and 0.75.
• mAP_S_, mAP_M_, and mAP_L_: segmentation accuracy for three scales of target fruits: small, medium, and large.
• Params: model parameters for measuring the simplicity.
• FLOPs: floating point of operations for measuring the complexity.
• FPS: frames per second, that is, the inference speed. The higher the value, the faster the processing speed.

### Result visualization

The fruit targets in the green apple dataset are split into three different scales (small, medium, and large) based on certain regional scopes. Compared with the clear and complete target fruits that exist independently in the image, it is relatively more difficult to recognize and segment green apples under multiple interference conditions, especially the fruits in small target regions. To this end, we first visualize the model for segmenting green apple fruits at different scales to verify the effectiveness for various green fruits.

**Fig. 8.**
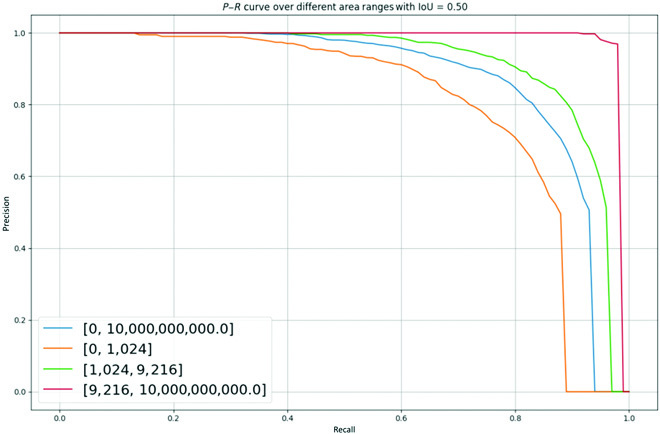
*P*–*R* curves of object fruits with various scales.

When the IoU threshold is set to 0.5, the *P*–*R* curves of the model for various fruit scales at a maximum number of recognition of 100 are shown in Fig. 8, where the yellow, green, and red lines stand for the *P*–*R* variation curves for small-, medium-, and large-scale fruits, respectively. It can be seen that the yellow line is overall lower than the other colors. This illustrates the fact that the model is generally more difficult to recognize for small target fruits and the segmentation accuracy is lower than the average (blue line). However, our model achieves a prediction accuracy of 0.7 when recall equals 0.8, which has a high potential and can be further exploited.

**Fig. 9.**
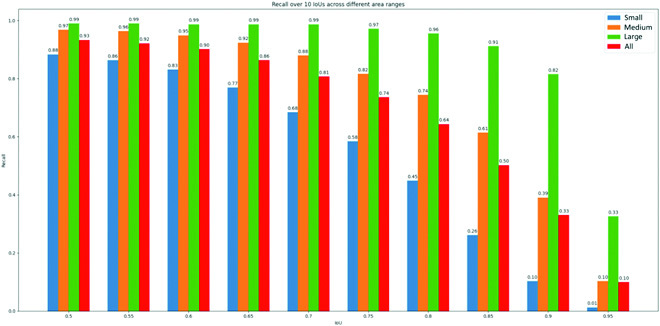
Recall for multiscale objects at 10 IoUs.

Moreover, Fig. 9 shows the recall of SE-COTR for multiscale object fruits at 10 IoU thresholds. The model has a high recall rate of close to 1 for green apple targets above medium size at IoU of 0.5, meaning that it recalls almost all of the larger fruits. Of most interest is the 88% recall rate for the recognition of small targets as well, suggesting that more effective improvements can be made to enhance the overall segmentation performance of the model.

### Ablation experiments

Ablation experiments are conducted on the structural branches of the model to verify the effectiveness of the main modules.

#### Backbone comparison

To understand the impact of the new lightweight backbone network on the efficiency of the model, we compare the capability of different backbone networks for extracting features with classic ResNet50+FPN backbone as a data reference. The results are shown in Table 2.

**Table 2.** Backbone comparison results.

**Backbone**	mAP/%	AP_50_/%	FPS
ResNet50 + FPN	58.7	83.1	12.4
**MobileNetV2** + FPN	59.4**(+0.7)**	84.6**(+1.5)**	20.5**(+8.1)**

After replacing only the backbone network with MobileNet, the mask accuracy is improved by about 0.7 point and 1.5 point in mAP and AP_50_, respectively, with more processing frames per second. Compared to ResNet, lightweight MobileNetV2 is able to extend the low-dimensional compressed representation of the input to higher dimensions, use lightweight deep convolution for filtering, and then project the features back to the low-dimensional compressed representation. This method of dimension enhancement and then dimension reduction can better preserve the information in the low-dimensional space and effectively transfer the information from low dimensional to high dimensional. As a result, MobileNetV2 is indeed able to extract abundant features, thus improving the localization accuracy and overall efficiency of the model.

#### Transformer type

To show the effectiveness of the COTR in processing features, we have compared the overall performance using the original model and replacing COTR only separately. The results for these variables are shown in Table 3. By using COTR to extract and process fruit features, better segmentation performance is achieved, especially in small fruit segmentation, which achieves 5.4 gain in mAP_S_ with much fewer parameters and FLOPs. The proposed COTR efficiently extracts valid features and greatly outperforms the original transformer in all metrics. This also illustrates adequately a fact. COTR structure successfully captures the spatial location relationship between horizontal and vertical coordinates. In addition, when combined with the CNN backbone, the features and representations of the images can be learned more efficiently.

**Table 3.** Comparisons of proposed COTR with original transformer.

**Transformer**	mAP_S_/%	Params/M	FLOPs/G
Original	36.3	52.7	220.8
COTR	41.7**(+5.4)**	43.2**(−9.5)**	192.5**(−28.3)**

### Model comparisons

After training on the same dataset, the performance of the model is tested using validation sample sets. Standard COCO metrics are recorded to clearly demonstrate the segmentation accuracy of the proposed model. The overall performance of the model is assessed for the completed trained network using the above metrics.

In addition, to verify the performance of the SE-COTR, we compared it with the start-of-art instance segmentation methods and analyzed the evaluation consequences on the green apple dataset to substantiate the validity of the proposed model. The model capacity and complexity are evaluated by Params and FLOPs, respectively, and the concrete consequences are shown in Table 4. The consequences suggest that the SE-COTR using MobileNetV2 backbone outperforms other segmentation techniques with the mAP of 61.6%, which is a substantial improvement.

**Table 4.** Comparisons with state-of-the-art models (input image size is set to 1,216 × 800).

**Model**	**mAP/%**	**AP_50_/%**	**mAP_S_/%**	**mAP_M_/%**	**mAP_L_/%**	**Params/M**	**FLOPs/G**	**FPS**
PointRend [36]	60.1	86.2	41.8	66.6	88.2	55.5	195.2	17.6
Mask R-CNN [37]	58.8	84.8	41.1	65.1	87.1	43.8	248.5	10.3
MS R-CNN [38]	58.4	84.7	40.7	64.0	86.9	60.0	248.5	12.8
YOLACT [39]	51.9	80.9	31.6	59.6	84.8	**34.7**	177.0	30.4
SE-COTR **(ours)**	**61.6**	**86.8**	**43.3**	**68.1**	**89.6**	36.2	**156.0**	**32.5**

Compared with other modern advanced instance segmentation methods, SE-COTR achieves better segmentation performance than other models on almost all other metrics, especially on the two-stage R-CNN series models. Specifically, SE-COTR keeps a lead in mAP, AP_50_, and other accuracy metrics compared to other state-of-the-art models, and the parameters and model complexity are substantially lower than most other algorithms. Although the number of SE-COTR parameters is slightly higher in comparison to YOLACT, the SE-COTR achieves a 9.7 gain in mAP and 11.7 gain in mAP_S_.

Taking into account model complexity, the metric FLOPs is applied for specific measurements. As listed in the Table 5, SE-COTR has the lowest complexity with a similar input size. For the general two-stage segmentation model like Mask R-CNN and Mask Scoring (MS) R-CNN, in addition to the basic backbone, neck, and prediction head, the region suggestion network and region of interest are also required to be calculated, which requires more FLOPs. Hence, as shown in Table 5, the models of the R-CNN series reach the highest complexity, while our SE-COTR has the lowest complexity as a similar input size compared to the popular segmentation models mentioned above. Besides, SE-COTR achieves higher FPS value at 32.5 frames per second and is obviously superior to other models in terms of segmentation speed, which means that SE-COTR has a more comprehensive and efficient performance in real-time fruit segmentation and recognition.

**Table 5.** Comparison with other fruit segmentation methods.

Methods	mAP/%	AP_50/_%	Params/M	FLOPs/G
YOLOF-Snake [40]	52.6	80.7	64.0	178.2
Improved FCOS /cba [41]	60.4	**87.3**	39.7	169.8
SE-COTR **(ours)**	**61.6**	86.8	**36.2**	**156.0**

**Fig. 10.**
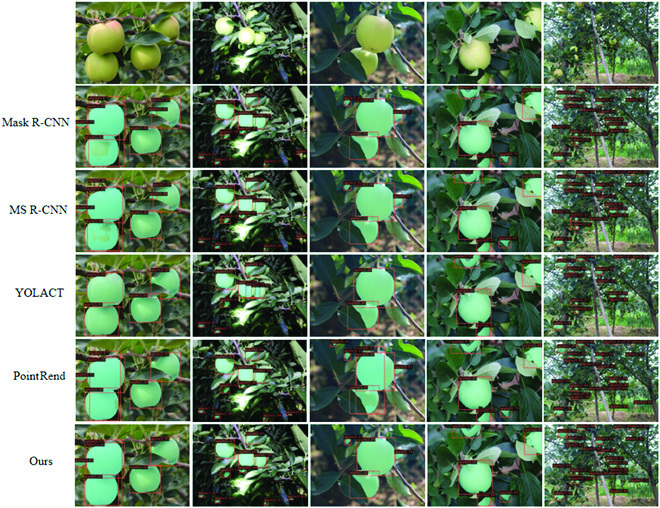
Segmentation results of SE-COTR and other techniques.

Moreover, illumination is one of the crucial factors to recognize the objects in computer vision. To further evaluate the model segmentation performance in complex orchard, we selected some green apple images under various interference conditions to visualize the model segmentation effect, including interference elements such as severe obscuring, variable lights, and highly dense fruit. Specifically, under the same experimental environment settings, consistent dataset processing, and uniform weights of all model parameters, we compared the fruit segmentation results of SE-COTR with popular Mask R-CNN, MS R-CNN, YOLACT, and PointRend models on the apple dataset and employed the same postprocessing to make fair comparisons. The segmentation consequences of these models for apple images can be seen in Fig. 10.

As shown in Fig. 10, comparing the visualized images of green apple segmentation in normal light, at night, in side light, after rain, and in distant view, it is apparent that our model outperforms other segmentation techniques for green apples in each scenario. Especially in the cases of overlapping fruits, branches and foliage occlusion, and inadequate illumination at night, the advantages of our method are more obvious. Figure 10 shows that even in the presence of severe interference, our segmentation model can accurately and directly recognize and segment the object fruit, achieving accurate and efficient green fruit segmentation. Specifically, the segmentation results of Mask R-CNN and MS R-CNN both have misdetection for overlapping green apple fruit. They make mistakes in distinguishing different instances of fruit and are prone to judge two heavily overlapping or obscured green fruits as the same instance. The inaccuracy of fruit counting will affect the application of the model for yield prediction of early immature fruits. PointRend locates and segments the presence of fruits very well, almost every fruit is successfully identified and segmented, but there is a phenomenon of false identification of green branches and leaves belonging to the background area as fruit targets, and excessive segmentation tends to be time consuming and computationally expensive. In addition, YOLACT suffers from obvious small target fruit omission and is prone to incorrectly detecting changing regions as additional fruit targets in a variable light environment, with poor robustness and segmentation accuracy.

The proposed SE-COTR effectively avoids the above-mentioned cases of missed and false detection and is able to accurately identify and segment on small-, medium-, and large-scale green apple targets. Furthermore, it has strong enough adaptability and robustness for fruit segmentation in complex and variable natural environments and achieves accurate and efficient fruit segmentation with fewer parameters and FLOPs than other segmentation models.

The segmentation result diagram includes the effect of fruit segmentation in the presence of occlusion, thus illustrating that accurate fruit masks can also be obtained for occluded fruit. The relatively low-resolution P5 feature maps output by COTR contain rich target location information, which helps the model to locate and recognize targets of interest more accurately, so that obscured fruit can also be accurately located. Specifically, the coordinate attention used in the COTR layer enhances the representation of the feature map by integrating the input features in the vertical and horizontal directions into the generated attention map, respectively, effectively capturing direction-aware and position-aware information. This encoding process allows more accurately position of the object of interest and hence helps the whole model to recognize better.

### Comparison with other fruit segmentation methods

To further analyze the application performance of the network, we compare the segmentation performance of the proposed SE-COTR with newest fruit segmentation methods on the green apple dataset that we created using the standard COCO metrics, as shown in Table 5.

In terms of accuracy, SE-COTR is far superior to the YOLOF-Snake fruit segmentation model constructed on the basis of the YOLOF detection model [34]. SE-COTR is substantially ahead in mAP and AP_50_ metrics and has lower parameters and model complexity than this model. Compared to the improved accurate segmentation model based on the single-stage detection model Fully Convolutional One-Stage (FCOS) [35], SE-COTR has a 1.2 improvement in the mAP and an advantage in the number of parameters and model complexity, although it is slightly lower than this model in the precision AP_50_ with an IoU threshold of 0.5. Thus, the overall performance of the SE-COTR fruit segmentation model is more accurate and simpler.

In summary, SE-COTR achieves accurate and stable target fruit segmentation performance for green apple fruits in complex orchard environments, which can meet the efficient requirements for robot working and can be migrated to other fruit detection and segmentation applications.

## Discussion

In this work, a novel fruit segmentation model SE-COTR is proposed to realize accurate fruit segmentation for green apples in the natural orchard, which is applicable for efficient real-time work of harvesting robots. With a lightweight mobile network as the backbone, the model is more flexible and better suited for embedding into vision systems for various agricultural intelligence applications. In addition, the coordinate attention mechanism is employed to build a COTR module to extract and fuse feature regions more clearly by gathering resembling features and curbing confusion caused by homochromatic branches and leaves. Furthermore, JPU module is employed to integrate features from each FPN layer and COTR into the uniform mask feature. Finally, on the basis of the outputs of the function heads, the dynamic convolution operation is used to generate the instance mask. The experiments indicate that SE-COTR is able to achieve accurate and efficient fruit segmentation with 61.6% mAP using fewer parameters and less FLOPs, which contributes to the real-time segmentation applications of various fruits and vegetables in complex environments.

The SE-COTR segmentation model proposed in this paper focuses on solving the challenge of green target fruit recognition in complex practical orchard environments, with high target fruit recognition accuracy and efficiency, to aid yield estimation and automated harvesting in orchards, in order to achieve intelligent management of orchards. During the construction of the SE-COTR model, the lightweight network design is used to improve the practicality and assemblability of the segmentation model.

In order to fit the orchard robot, we intend to use an “edge device + camera + solar/battery” model. The edge computing device is an NVIDIA Jetson Nano, which facilitates the deployment of segmentation algorithms on the device; the camera can be selected according to the actual needs of the orchard; and the power supply module is a combination of solar/battery in order to improve the endurance of the device. During the hardware equipment assembly process, industrial cameras of smaller size and weight are being considered in order to better fit the edge equipment and the robot body. The relatively lightweight Microvision series of working cameras with a resolution that is adequate for target fruit recognition is currently being commissioned. The next step in our research focuses on pairing segmentation models on hardware to test the algorithms and equipment performance in the natural orchard operating environments.

## Data Availability

Anyone who wants to use the data can contact the corresponding author W.J. The author is with the School of Information Science and Engineering, Shandong Normal University, Jinan 250358, China (e-mail: jwk_1982@163.com).

## References

[1] Bauer A, Bostrom AG, Ball J, Applegate C, Cheng T, Laycock S, Rojas SM, Kirwan J, Zhou J. Combining computer vision and deep learning to enable ultra-scale aerial phenotyping and precision agriculture: A case study of lettuce production. Hortic Res. 2019;6(1):70. 31231528 10.1038/s41438-019-0151-5 PMC6544649

[2] Stein M, Bargoti S, Underwood J. Image based mango fruit detection, localisation and yield estimation using multiple view geometry. Sensors. 2016;16(11):1915. 27854271 10.3390/s16111915 PMC5134574

[3] Jia W, Zhang Y, Lian J, Zheng Y, Zhao D, Li C. Apple harvesting robot under information technology: A review. Int J Adv Robot Syst. 2020;17(3):1729881420925310.

[4] Fan P, Lang G, Yan B, Lei X, Guo P, Liu Z, Yang F. A method of segmenting apples based on gray-centered RGB color space. Remote Sens. 2021;13(6):1211.

[5] Wang Z-F, Jia W-K, Mou S-H, Hou S-J, Yin X, Ze J. KDC: A green apple segmentation method. Spectrosc Spectr Anal. 2021;41(9):2980–2988.

[6] Liu X, Jia W, Ruan C, Zhao D, Gu Y, Chen W. The recognition of apple fruits in plastic bags based on block classification. Precis Agric. 2018;19(4):735–749.

[7] Kang H, Chen C. Fruit detection and segmentation for apple harvesting using visual sensor in orchards. Sensors. 2019;19(20):4599. 31652634 10.3390/s19204599 PMC6832306

[8] Kong T, Sun F, Liu H, Jiang Y, Li L, Shi J. Foveabox: Beyound anchor-based object detection. IEEE Trans Image Process. 2020;29:7389–7398.

[9] Zhang S, Chi C, Yao Y, Lei Z, Li SZ. Bridging the gap between anchor-based and anchor-free detection via adaptive training sample selection. Paper presented at: Proceedings of the IEEE/CVF Conference on Computer Vision and Pattern Recognition; 2020 June 14–19; Seattle. p. 9759–9768.

[10] Kamilaris A, Prenafeta-Boldú FX. Deep learning in agriculture: A survey. Comput Electron Agric. 2018;147:70–90.

[11] Jia W, Wang Z, Zhang Z, Yang X, Hou S, Zheng Y. A fast and efficient green apple object detection model based on Foveabox. J King Saud Univ - Comput Inf Sci. 2022;34(8):5156–5169.

[12] Bargoti S, Underwood JP. Image segmentation for fruit detection and yield estimation in apple orchards. J Field Robot. 2017;34(6):1039–1060.

[13] Kim J, Seol J, Lee S, Hong S-W, Son HI. An intelligent spraying system with deep learning-based semantic segmentation of fruit trees in orchards. Paper presented at: 2020 IEEE International Conference on Robotics and Automation (ICRA). IEEE, 2020 May 31–Aug 31; Paris, France. p. 3923–3929.

[14] Jia W, Tian Y, Luo R, Zhang Z, Lian J, Zheng Y. Detection and segmentation of overlapped fruits based on optimized mask R-CNN application in apple harvesting robot. Comput Electron Agric. 2020;172:105380.

[15] Liu J, Zhao Y, Jia W, Ji Z. DLNet: Accurate segmentation of green fruit in obscured environments. J King Saud Univ - Comput Inf Sci. 2021;34(9):7259–7270.

[16] Jia W, Zhang Z, Shao W, Ji Z, Hou S. RS-Net: Robust segmentation of green overlapped apples. Precis Agric. 2021;23:492–513.

[17] Li Q, Jia W, Sun M, Hou S, Zheng Y. A novel green apple segmentation algorithm based on ensemble U-Net under complex orchard environment. Comput Electron Agric. 2021;180:105900.

[18] Hartley ZKJ, Jackson AS, Pound M, French AP. GANana: Unsupervised domain adaptation for volumetric regression of fruit. Plant Phenomics. 2021;9874597. 34708214 10.34133/2021/9874597 PMC8520669

[19] Wang X, Zhang R, Kong T, Li L, Shen C. Solov2: Dynamic and fast instance segmentation. Adv Neural Inf Proces Syst. 2020;33:17721–17732.

[20] Tsotsos JK. *A computational perspective on visual attention*. MIT Press: Cambridge, Massachussetts; 2011.

[21] Bello I, Zoph B, Le Q, Vaswani A, Shlens J. Attention augmented convolutional networks. Paper presented at: Proceedings of the IEEE/CVF International Conference on Computer Vision; 2019 Oct 27–Nov 2; Seoul, Korea (South). p. 3286–3295.

[22] Hou Q, Zhang L, Cheng MM, Feng J. Strip pooling: Rethinking spatial pooling for scene parsing. Paper presented at: Proceedings of the IEEE/CVF Conference on Computer Vision and Pattern Recognition; 2020 June 14–19; Seattle. p. 4003–4012.

[23] He K, Zhang X, Ren S, J Sun. Deep residual learning for image recognition. Paper presented at: Proceedings of the IEEE Conference on Computer Vision and Pattern Recognition; 2016 June 27–30; Las Vegas, NV, USA. p. 770–778.

[24] Simonyan K, Zisserman A. Very deep convolutional networks for large-scale image recognition. arXiv. 2014. 10.48550/arXiv.1409.1556

[25] Guo R, Niu D, Qu L, Li Z. Sotr: Segmenting objects with transformers. Paper presented at: Proceedings of the IEEE/CVF International Conference on Computer Vision; 2021 Oct. 10–17; Montreal, Canada. p. 7157–7166.

[26] Sandler M, Howard A, Zhu M, Zhmoginov A, Chen L-C. Mobilenetv2: Inverted residuals and linear bottlenecks. Paper presented at: Proceedings of the IEEE Conference on Computer Vision and Pattern Recognition; 2018 June 18–23; Salt Lake City. p. 4510–4520.

[27] Lin TY, Dollár P, Girshick R, He K, Hariharan B, Belongie S. Feature pyramid networks for object detection. Paper presented at: Proceedings of the IEEE Conference on Computer Vision and Pattern Recognition; 2017 July 21–26; Honolulu. p. 2117–2125.

[28] Hou Q, Zhou D, Feng J. Coordinate attention for efficient mobile network design. Paper presented at: Proceedings of the IEEE/CVF Conference on Computer Vision and Pattern Recognition; 2021 June 20–25; Nashville, TN, USA. p. 13713–13722.

[29] Wu H, Zhang J, Huang K, Liang K, Yu Y. Fastfcn: Rethinking dilated convolution in the backbone for semantic segmentation. arXiv. 2019. 10.48550/arXiv.1903.11816

[30] He K, Zhang X, Ren S, Sun J. Identity mappings in deep residual networks. Eur Conf Comput Vis. 2016;9908:630–645.

[31] Redmon J, Divvala S, Girshick R, Farhadi A. You only look once: Unified, real-time object detection. Paper presented at: Proceedings of the IEEE Conference on Computer Vision and Pattern Recognition; 2016 June 27–30; Las Vegas, NV, USA. p. 779–788.

[32] Milletari F, Navab N, Ahmadi SA. V-net: Fully convolutional neural networks for volumetric medical image segmentation. Paper presented at: 2016 Fourth International Conference on 3D Vision (3DV). IEEE; 2016 October 25–28; Stanford, CA, USA. p. 565–571.

[33] Lin TY, Maire M, Belongie S, Hays J, Perona P, Ramanan D, Dollár P, Zitnick CL. Microsoft coco: Common objects in context. In: Fleet, D, Pajdla, T, Schiele, B, Tuytelaars, T, editors. *European conference on computer vision*. Cham: Springer; 2014. p. 740–755.

[34] Chen Q, Wang Y, Yang T, Zhang X, Cheng J, Sun J. You only look one-level feature. Paper presented at: Proceedings of the IEEE/CVF Conference on Computer Vision and Pattern Recognition; 2021 June 18–24; Nashville, TN. p. 13039–13048.

[35] Tian Z, Shen C, Chen H, T He. Fcos: Fully convolutional one-stage object detection. Paper presented at: Proceedings of the IEEE/CVF International Conference on Computer Vision; 2019 Oct. 27–Nov. 2; Seoul, Korea. p. 9627–9636.

[36] Kirillov A, Wu Y, He K, Girshick R. Pointrend: Image segmentation as rendering. Paper presented at: Proceedings of the IEEE/CVF Conference on Computer Vision and Pattern Recognition; 2020 June 13–19; Seattle, WA. p. 9799–9808.

[37] He K, Gkioxari G, Dollár P, Girshick R. Mask r-cnn. Paper presented at: Proceedings of the IEEE International Conference on Computer Vision; 2017 Oct 22–29; Venice, Italy. p. 2961–2969.

[38] Huang Z, Huang L, Gong Y, Huang C, Wang X. Mask scoring r-cnn. Paper presented at: Proceedings of the IEEE/CVF Conference on Computer Vision and Pattern Recognition; 2019 June 15–20; Long Beach, CA. p. 6409–6418.

[39] Bolya D, Zhou C, Xiao F, Lee YJ. Yolact: Real-time instance segmentation. Paper presented at: Proceedings of the IEEE/CVF International Conference on Computer Vision. 2019 Oct 27–Nov 2; Seoul, Korea (South). p. 9157–9166.

[40] Jia W, Liu M, Luo R, Wang C, Pan N, Yang X, Ge X. YOLOF-Snake: An efficient segmentation model for green object fruit. Front Plant Sci. 2022;13:765523. 35755692 10.3389/fpls.2022.765523 PMC9218684

[41] Liu M, Jia W, Wang Z, Niu Y, Yang X, Ruan C. An accurate detection and segmentation model of obscured green fruits. Comput Electron Agric. 2022;197:106984.

